# Public Perceptions of AI in Medicine and Implications for Future Medical Education: Cross-Sectional Survey

**DOI:** 10.2196/89123

**Published:** 2026-04-15

**Authors:** Michael Constantin Kirchberger

**Affiliations:** 1 Dermatology Center Ingolstadt, Schlüterstr. 3a, Ingolstadt, 85057, Germany, 49 841 4936936; 2 Department of Dermatology, University Hospital Erlangen, German Center for Immunotherapy, Friedrich-Alexander University Erlangen-Nuremberg, Erlangen, Germany

**Keywords:** artificial intelligence, AI, medical education, public perception, survey, trust, curriculum, physician training

## Abstract

**Background:**

The integration of artificial intelligence (AI) into clinical practice is contingent on public trust. This trust often depends on physician oversight, yet a significant gap exists between the need for AI-competent physicians and the current state of medical education. While the perspectives of students and experts on this gap are known, the views of the US general public remain largely unquantified.

**Objective:**

This study aimed to assess US public perceptions regarding AI in medicine and the corresponding emergent needs for medical education. We specifically sought to quantify public trust in different diagnostic scenarios, concerns about physician overreliance on AI, support for mandatory AI education, and priorities for the future focus of medical training.

**Methods:**

We conducted a cross-sectional, web-based survey of adults in the United States in November 2025. Participants (N=524) were recruited via SurveyMonkey Audience. We calculated descriptive statistics, frequencies, proportions (percentages), and 95% CIs for all main survey items.

**Results:**

A total of 524 participants completed the survey. Most (n=329, 62.8%; 95% CI 58.6%‐66.9%) placed the most trust in a physician’s diagnosis based on their expertise alone; only 7.8% (n=41; 95% CI 5.5%‐10.1%) trusted an AI-first diagnostic model. Trust was highly contingent on training: 93.9% (n=492) of participants rated formal physician training on AI limitations as “essential” or “very important.” Widespread concern about physician overreliance on AI was reported, with 81.1% (n=425) being “very concerned” or “extremely concerned.” Consequently, 85.1% (n=446) agreed or strongly agreed that training on AI use, ethics, and limitations should be mandatory in medical school. When asked about future educational priorities, 70.2% (n=368; 95% CI 66.3%‐74.1%) believed that medical education should focus on human-centered skills (eg, empathy and communication) over clinical skills.

**Conclusions:**

The US public expressed conditional trust in medical AI, strongly preferring physician-led and critically supervised models. These findings reveal a clear public mandate for medical education reform. The public expects future physicians to be mandatorily trained to appraise AI, understand its limitations, and refocus their professional development on the human-centered skills that technology cannot replace.

## Introduction

The integration of artificial intelligence (AI) into clinical practice represents a significant paradigm shift in health care. While public awareness of AI in general is growing, understanding of its concrete applications in medicine, such as diagnostics or treatment, remains substantially lower [1]. As these technologies move from development to deployment, their acceptance hinges on a critical and complex challenge: patient trust.

Current evidence on patient trust presents a conflicted picture. Some US-based experiments indicate that patients may have a strong baseline preference for human-only care, demonstrating a significant AI trust gap where trust in a physician decreases when AI is used [2]. However, other large-scale US studies have found that the public is almost evenly split in their preference between a human specialist and an “AI clinic” [3].

This complex trust dynamic appears tied not to the technology itself but to its governance and the role of the human operator. Public attitudes are strongly driven by demands for high reliability and transparency [4], and qualitative studies confirm that “fear of insufficient human oversight” and a “need for training of end users” are key barriers to acceptance [5]. To improve patient acceptance of automated technology, recent research emphasizes the need to design for an “appropriate level of trust,” ensuring that users understand how ambiguous clinical situations can challenge trust in AI outputs [6]. This places a heavy emphasis on the physician’s ability to critically evaluate and safely implement AI tools, a competence that must be built through formal education.

However, a significant gap exists between this emergent need and the current state of medical training. Studies of physicians and medical students report low familiarity with AI concepts, with very few having taken any formal AI courses [7]. This gap is starkly illustrated in US medical schools, where over 91% of students believe that training on AI would be useful for their careers, yet an identical proportion (91%) either report that their schools do not offer such resources or are unsure of whether they exist [8]. This lack of formal education is systemic; in a 2025 survey of US osteopathic medical schools, 93% of deans reported having no student-focused generative AI policy, and among colleges without a policy, 79% had no formal plans for mandatory student training, and 73% had no plans for elective training [9]. Leaving students unprepared is especially risky given the dual nature of generative AI in medical education: while it can enhance learning, it also threatens to undermine cognitive autonomy if trainees internalize biased, inaccurate, or hallucinated content without expert guidance [10].

This creates a critical mismatch: the public’s trust is contingent on physician oversight, yet physicians are not being systematically trained for this role. While the perspectives of students [8,11] and educational experts [2,12] on this gap are well documented, the views of the general public remain largely unquantified. It is unknown how the public perceives this training gap, whether they support educational mandates, or how they believe AI should reshape the priorities of medical education.

To address these gaps, this study was designed to assess the perceptions of the US general public regarding the intersection of AI, medicine, and medical education. Our primary objectives were to (1) assess public trust in different human-AI diagnostic scenarios, (2) quantify public concern about physician overreliance on AI, (3) measure public support for mandatory training on AI in medical schools, and (4) elicit public priorities for the future focus of medical education (human-centered vs clinical skills).

## Methods

### Study Design and Setting

We conducted a cross-sectional, descriptive study using a web-based survey. This study was designed to assess the perceptions of the general public in the United States regarding the role of AI in medicine and the corresponding needs for medical education. The survey was administered online in November 2025.

### Participant Recruitment and Sampling

Participants were recruited from the United States using SurveyMonkey Audience (SurveyMonkey Inc), a commercial panel that provides access to a nonprobability, convenience sample of respondents. The survey was targeted at the general adult population. Inclusion criteria for participation were (1) being aged 18 years or older, (2) residing in the United States, and (3) providing digital informed consent to participate. Recruitment was systematically managed through the SurveyMonkey platform, which draws from a diverse pool of opted-in individuals who participate via partner panels or the SurveyMonkey Contribute program. On the basis of our defined inclusion criteria, the platform automatically routed eligible users to our web-based survey.

### Survey Instrument

The survey instrument was a 12-item questionnaire developed by the study author comprising 7 main items and 5 demographic items. The main questions assessed several key domains: respondent trust in different diagnostic scenarios (eg, physician only and AI as a “second opinion”); perceptions on AI training and governance, including the importance of training on AI limitations, concerns about physician overreliance on AI, and mandatory AI education in medical schools; and ratings of physicians’ current digital skills. The instrument also queried about opinions on the future focus of medical education (human-centered vs clinical skills) and respondents’ experiences with physician reactions to patient-initiated discussions about AI-derived health information. Demographic data collected included age, gender, annual household income, and major US region. All questions were presented in a multiple-choice format.

### Data Analysis

All analyses were descriptive in nature. Initial data processing and frequency calculations were performed using SurveyMonkey’s built-in analysis tools. Data were then exported for further analysis. We calculated frequencies and proportions (percentages) for all categorical variables from the main survey questions and demographic items. The final analysis and data handling were conducted using Python (version 3.13; Python Software Foundation). Given the descriptive, cross-sectional design, no inferential statistical tests for causation were performed. For all proportions, 95% CIs were calculated using the normal approximation to the binomial distribution.

### Ethical Considerations

This study was conducted in accordance with the Declaration of Helsinki ethical principles for research involving human participants. All participants were presented with a digital information sheet on the survey’s landing page, which detailed the study’s purpose, the voluntary nature of participation, and the anonymity of their responses. Consent was obtained digitally before participants could proceed to the questionnaire. No personally identifiable information was collected, ensuring participant anonymity. Participants were recruited via SurveyMonkey and compensated through the platform’s standard incentive scheme.

According to the Professional Code for Physicians in Bavaria [13], research projects involving strictly anonymized data are not subject to mandatory ethical consultation. Consequently, this study was exempt from formal institutional review board review given the anonymous nature of the data and the minimal risk posed to participants (involving opinions rather than sensitive personal health data).

## Results

### Participant Demographics

A total of 524 individuals completed the main survey questions. While 99.2% (n=520) of these participants provided data on age, gender, income, and device type, slightly fewer (n=516, 98.5%) provided their US region. The participant characteristics are detailed in Table 1.

**Table 1.** Characteristics of the survey participants (n=520).

Characteristics	Participants, n (%)
Age group (y)	
18-29	23 (4.4)
30-44	196 (37.7)
45-60	210 (40.4)
>60	91 (17.5)
Sex	
Male	340 (65.4)
Female	180 (34.6)
Household income (US $)	
0-9999	24 (4.6)
10,000-24,999	26 (5.0)
25,000-49,999	56 (10.8)
50,000-74,999	47 (9.0)
75,000-99,999	33 (6.3)
100,000-124,999	52 (10.0)
125,000-149,999	77 (14.8)
150,000-174,999	55 (10.6)
175,000-199,999	79 (15.2)
≥200,000	50 (9.6)
Prefer not to answer	21 (4.0)

Most respondents (340/520, 65.4%) were male. The most common age groups were 45 to 60 years (210/520, 40.4%) and 30 to 44 years (196/520, 37.7%). Respondents were predominantly from the Pacific (190/516, 36.8%) and Mid-Atlantic (122/516, 23.6%) regions. The cohort represented a wide range of annual household incomes, with the most frequent brackets being US $175,000 to US $199,999 (79/520, 15.2%) and US $125,000 to US $149,999 (77/520, 14.8%). The vast majority of participants (499/520, 96.0%) responded via a mobile device, with Android phones or tablets being the most common (335/520, 64.4%).

### Trust in Diagnostic Scenarios

Participants were first asked the following question: “If you needed a complex diagnosis, which of the following scenarios would you trust the most?”

**Figure 1.**
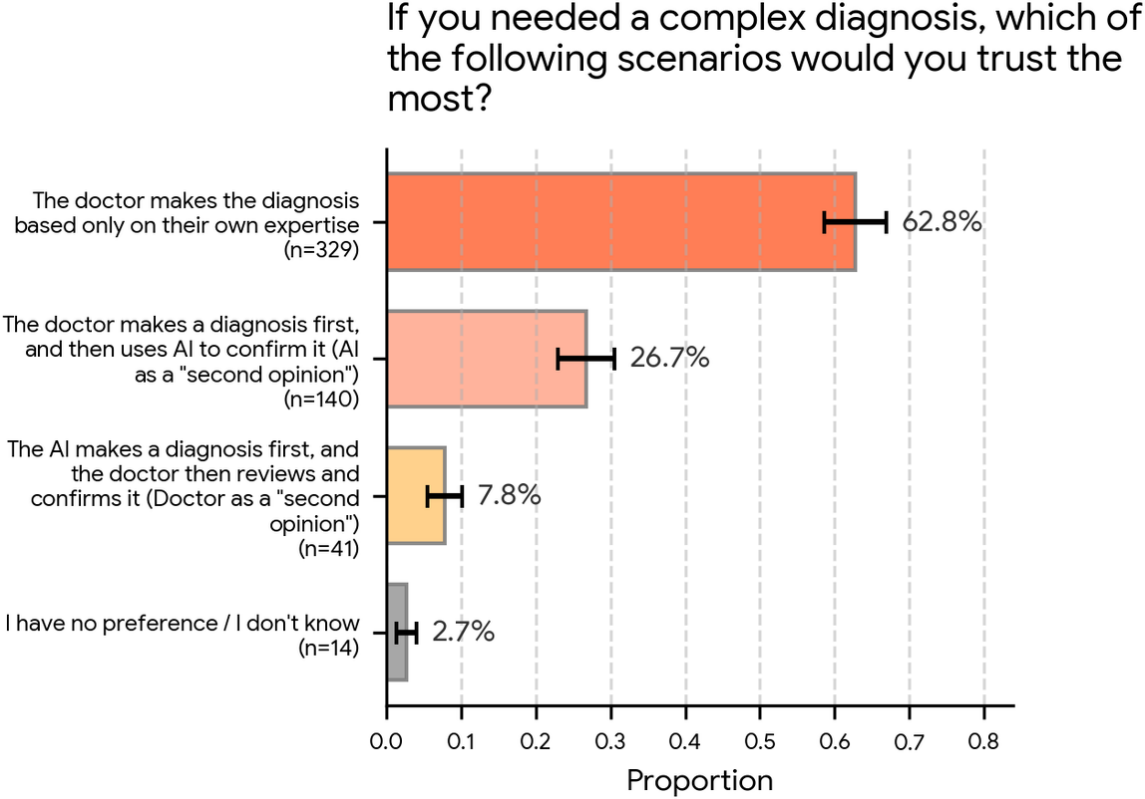
Public trust in different diagnostic scenarios involving physicians and artificial intelligence (AI; N=524). When asked which scenario they would trust most for a complex diagnosis, most (329/524, 62.8%) selected the physician’s diagnosis based on their expertise alone. The next most trusted scenario was the physician making the diagnosis first and then using AI to confirm it (140/524, 26.7%). The scenario in which AI makes the initial diagnosis and the physician reviews it was trusted the least (41/524, 7.8%). Error bars represent 95% CIs.

As shown in Figure 1, the vast majority of respondents (329/524, 62.8%; 95% CI 58.6%‐66.9%) selected the option “The doctor makes the diagnosis based only on their own expertise.” The second most common response was “The doctor makes a diagnosis first, and then uses AI to confirm it (AI as a ‘second opinion’),” chosen by 26.7% (140/524; 95% CI 22.9%‐30.5%) of the participants. Substantially fewer participants opted for the inverse scenario, “The AI makes a diagnosis first, and the doctor then reviews and confirms it (Doctor as a ‘second opinion’),” at 7.8% (41/524; 95% CI 5.5%‐10.1%). A small fraction (14/524, 2.7%; 95% CI 1.3%‐4.1%) selected “I have no preference/I don’t know.”

### Perceived Importance of Physician Training on AI Limitations

To gauge expectations regarding physician competency, participants were asked the following: “How important is it for your personal trust in a doctor’s decision, to know that the doctor has been formally trained to understand the limitations and potential errors of the AI systems they use?”

**Figure 2.**
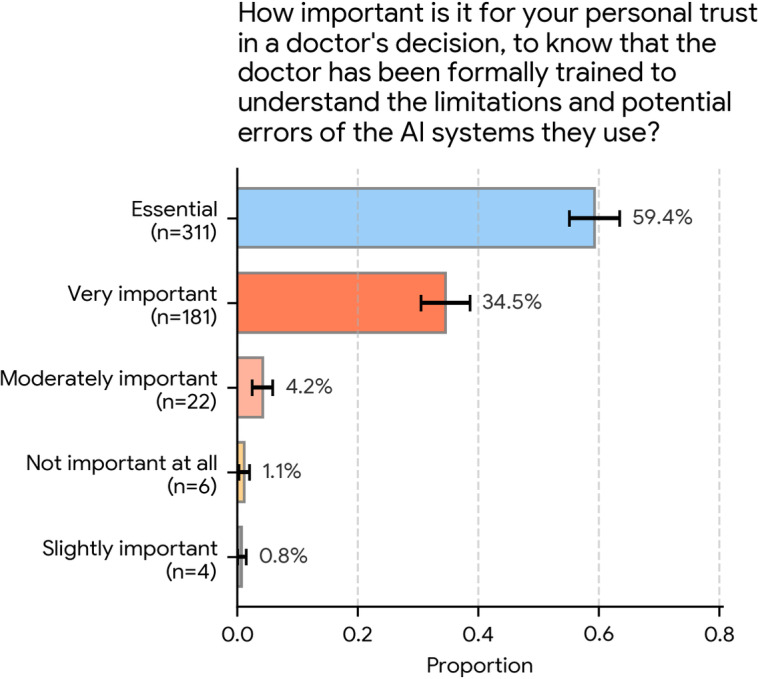
Perceived importance of formal physician training on artificial intelligence (AI) limitations (N=524). Respondents rated the importance of a physician being formally trained to understand the limitations and potential errors of the AI systems they use. An overwhelming cumulative majority of 93.9% (492/524) rated this training as “essential” (311/524, 59.4%) or “very important” (181/524, 34.5%) for their personal trust in the physician’s decision. Error bars represent 95% CIs.

The responses, detailed in Figure 2, indicate that such training is overwhelmingly viewed as critical for patient trust. A vast majority of respondents rated this training as either “essential” (311/524, 59.4%; 95% CI 55.1%‐63.6%) or “very important” (181/524, 34.5%; 95% CI 30.5%‐38.6%).

Together, 93.9% (492/524) of the participants considered this formal training to be at least “very important.” In contrast, very few respondents rated it as “moderately important” (22/524, 4.2%; 95% CI 2.5%‐5.9%), “slightly important” (4/524, 0.8%; 95% CI 0.0%‐1.5%), or “not important at all” (6/524, 1.1%; 95% CI 0.2%‐2.1%).

### Concern About Physician Overreliance on AI

The survey also explored public concern about the potential for physician overreliance on AI. Participants were asked the following: “How concerned are you that future doctors might rely too much on AI for diagnosing patients, without critically questioning the AI’s results?”

**Figure 3.**
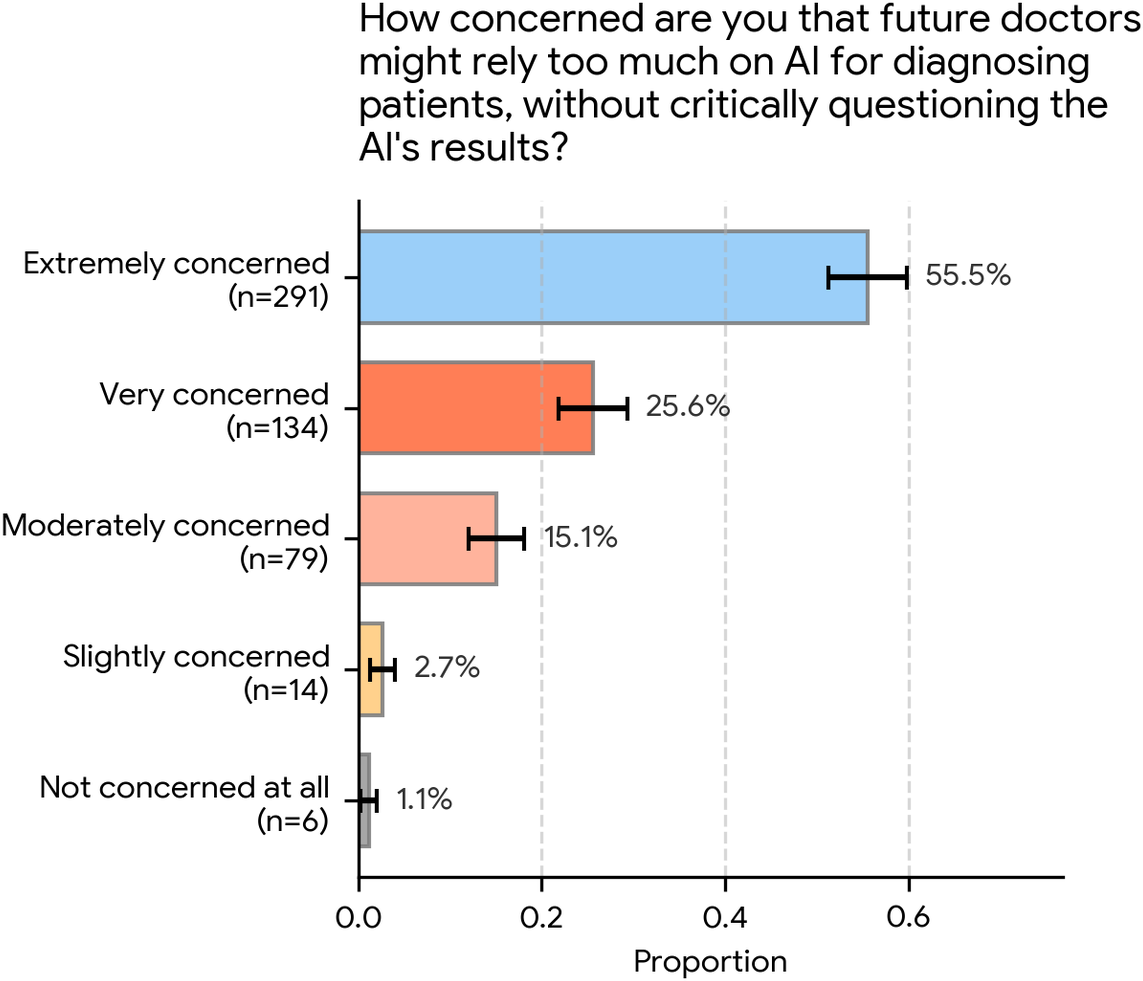
Public concern regarding future physician overreliance on artificial intelligence (AI; N=524). Participants rated their concern that future physicians might rely too much on AI results without critical questioning. A cumulative total of 81.1% (425/524) of the participants reported being “extremely concerned” (291/524, 55.5%) or “very concerned” (134/524, 25.6%) about this potential overreliance.

As Figure 3 illustrates, concern was widespread and strong. Most respondents (291/524, 55.5%; 95% CI 51.3%‐59.8%) reported being “extremely concerned,” and another 25.6% (134/524; 95% CI 21.8%‐29.3%) reported being “very concerned.”

Cumulatively, 81.1% (425/524) of the participants were “very concerned” or “extremely concerned” about this issue. A smaller group expressed moderate concern (79/524, 15.1%; 95% CI 12.0%‐18.1%), whereas very few were “slightly concerned” (14/524, 2.7%; 95% CI 1.3%‐4.1%) or “not concerned at all” (6/524, 1.1%; 95% CI 0.2%‐2.1%).

### Support for Mandatory AI Education in Medical Schools

To connect public concern with actionable policy, the survey asked participants the following: “To what extent do you agree or disagree with the following statement: ‘Training on the use, ethics, and limitations of Artificial Intelligence should be a mandatory (required) part of medical school education.’”

**Figure 4.**
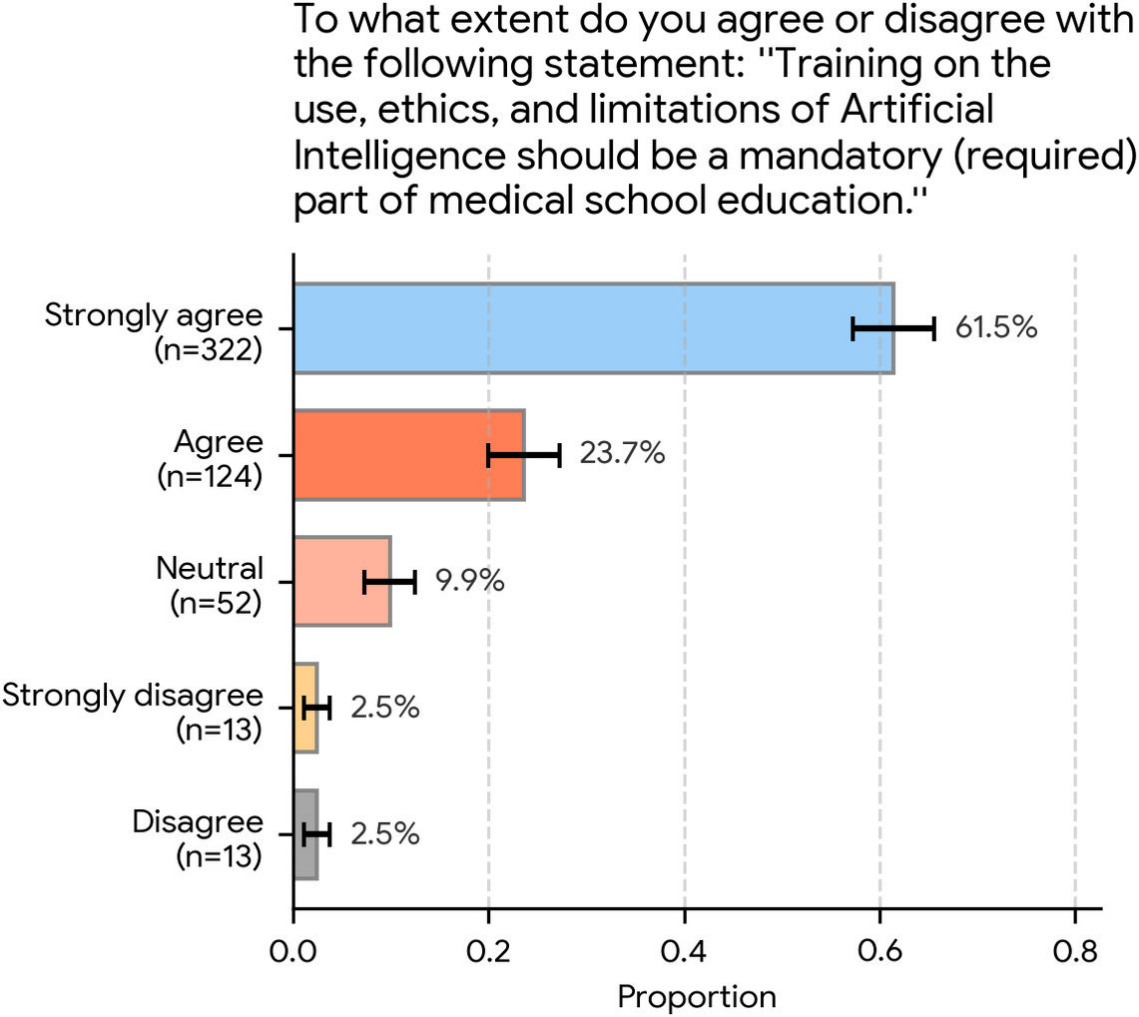
Support for mandatory artificial intelligence (AI) education in medical schools (N=524). Participants overwhelmingly agreed that training on the use, ethics, and limitations of AI should be a mandatory part of medical school education. A combined 85.1% (446/524) agreed or strongly agreed, with 61.5% (322/524) strongly agreeing. Error bars represent 95% CIs.

As shown in Figure 4, there was overwhelming agreement with this statement. Most participants (322/524, 61.5%; 95% CI 57.3%‐65.6%) selected “strongly agree,” and an additional 23.7% (124/524; 95% CI 20.0%‐27.3%) selected “agree.”

Combined, 85.1% (446/524) of all respondents agreed or strongly agreed that this training should be mandatory. A small portion remained “neutral” (52/524, 9.9%; 95% CI 7.4%‐12.5%), whereas only 5.0% (26/524) in total selected either “disagree” (13/524, 2.5%; 95% CI 1.1%‐3.8%) or “strongly disagree” (13/524, 2.5%; 95% CI 1.1%‐3.8%).

### Public Perception of Physicians’ Current Digital Skills

Participants were asked to rate their perception of physicians’ current digital proficiency using the following question: “Based on your general experience with the healthcare system, how would you rate the current digital skills of most doctors (e.g., using electronic health records, video consultations, or patient data from apps)?”

**Figure 5.**
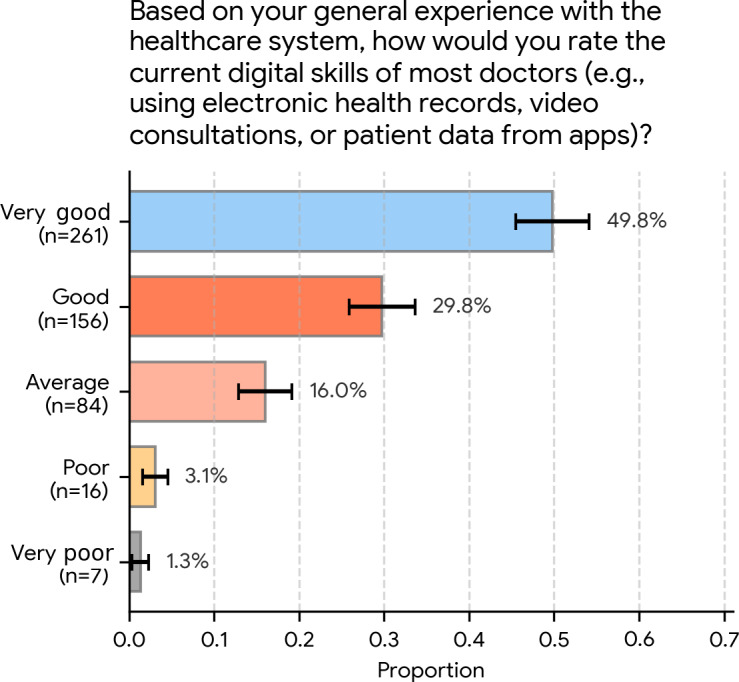
Public perception of the current digital skills of most physicians (N=524). Respondents rated the digital skills of most physicians (eg, using electronic health records or patient data from apps). A positive view was held by nearly 80% (417/524, 79.6%), rating physicians’ skills as “very good” (261/524, 49.8%) or “good” (156/524, 29.8%). Error bars represent 95% CIs.

The responses, shown in Figure 5, were generally positive. Just under half (261/524, 49.8%; 95% CI 45.5%‐54.1%) of the participants rated physicians’ skills as “very good,” and 29.8% (156/524; 95% CI 25.9%‐33.7%) rated them as “good.”

Collectively, nearly 80% (417/524, 79.6%) of respondents held a positive view (“good” or “very good”) of physicians’ digital skills. A smaller group rated them as “average” (84/524, 16.0%; 95% CI 12.9%‐19.2%). Very few participants perceived these skills as “poor” (16/524, 3.1%; 95% CI 1.6%‐4.5%) or “very poor” (7/524, 1.3%; 95% CI 0.4%‐2.3%).

### Future Priorities for Medical Education

Participants were asked to make a direct choice about the future focus of medical training using the following question: “Assuming AI will reliably handle more technical tasks (like analyzing scans) in the future, where should medical education place its primary focus to train better doctors?”

**Figure 6.**
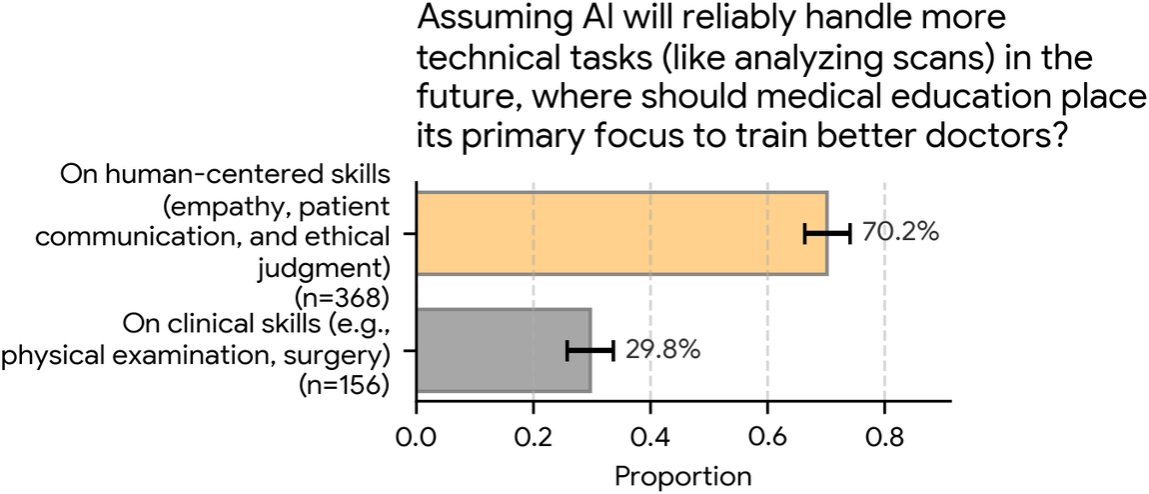
Priorities for future medical education focus (N=524). Assuming that artificial intelligence (AI) will reliably handle technical tasks (such as analyzing scans) in the future, participants were asked where medical education should focus. A large majority (368/524, 70.2%) prioritized “human-centered skills (empathy, patient communication, and ethical judgment)” over “clinical skills” (156/524, 29.8%). Error bars represent 95% CIs.

As Figure 6 shows, the response was a decisive, binary choice. A large majority of respondents (368/524, 70.2%; 95% CI 66.3%‐74.1%) believed that the primary focus should be “On human-centered skills (empathy, patient communication, and ethical judgment).”

Conversely, 29.8% (156/524; 95% CI 25.9%‐33.7%) believed that the focus should remain “On clinical skills (e.g., physical examination, surgery).”

### Patient-Reported Physician Reactions to AI-Informed Discussions

Finally, the survey assessed participants’ real-world experiences by asking the following: “Thinking about that consultation when you discussed AI information on symptoms, which of the following examples best describes how your doctor reacted?”

**Figure 7.**
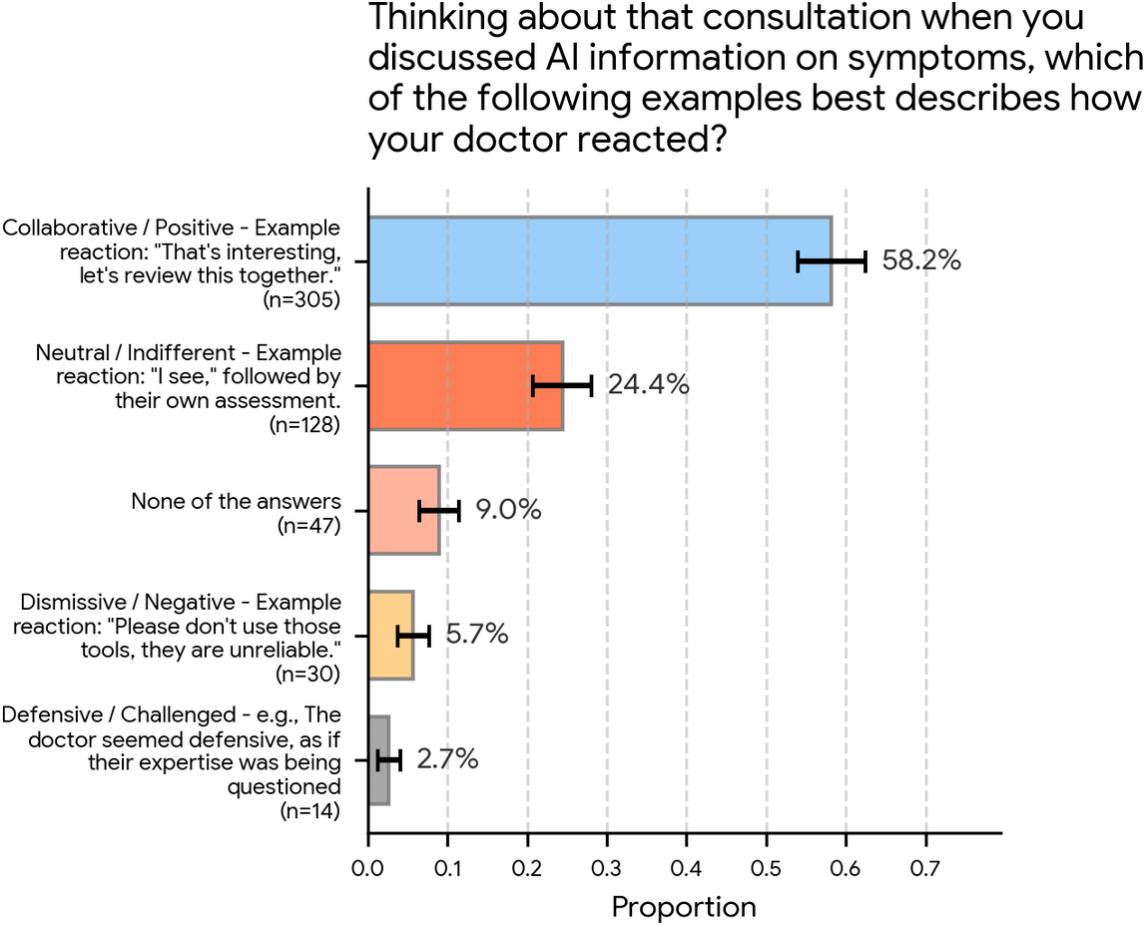
Patient-reported physician reactions when discussing artificial intelligence (AI) information on symptoms (N=524). Participants reported how their physician reacted during a consultation when information derived from AI regarding symptoms was discussed. The most common reaction was “Collaborative / Positive” (305/524, 58.2%), followed by a “Neutral / Indifferent” reaction (128/524, 24.4%). Negative or defensive reactions were reported by small minorities. Error bars represent 95% CIs.

As Figure 7 details, the most commonly reported reaction was positive. Most participants (305/524, 58.2%; 95% CI 54.0%‐62.4%) selected the “Collaborative / Positive” response, exemplified by “That’s interesting, let’s review this together.”

The second most frequent reaction was “Neutral / Indifferent” (eg, “I see” followed by their own assessment), reported by 24.4% (128/524; 95% CI 20.7%‐28.1%). A smaller group of 9.0% (47/524; 95% CI 6.5%‐11.4%) indicated that “None of the answers” fit their experience.

Negative reactions were the least common. Only 5.7% (30/524; 95% CI 3.7%‐7.7%) reported a “Dismissive/Negative” reaction (eg, “Please don’t use those tools, they are unreliable”), and 2.7% (14/524; 95% CI 1.3%‐4.1%) reported a “Defensive/Challenged” reaction.

## Discussion

### Principal Findings

In this national web-based survey of adults in the United States, respondents expressed a clear preference for physician-led diagnosis in complex clinical scenarios. Almost two-thirds (329/524, 62.8%; 95% CI 58.6%‐66.9%) indicated highest trust in a scenario in which the physician made a diagnosis based only on their own expertise. In contrast, only approximately one-quarter preferred a model where the physician made the initial diagnosis and then consulted AI as a “second opinion” (140/524, 26.7%; 95% CI 22.9%‐30.5%). Very few respondents favored AI making the first diagnosis with a physician as the checker (41/524, 7.8%; 95% CI 5.5%‐10.1%).

At the same time, trust in physicians’ use of AI was strongly contingent on formal training. An overwhelming majority (492/524, 93.9%) rated training on AI limitations and potential errors as at least “very important” for their personal trust. More than four-fifths (425/524, 81.1%) reported being “very concerned” or “extremely concerned” that future physicians might rely too heavily on AI. Consequently, most respondents (446/524, 85.1%) supported mandatory AI education in medical schools. Respondents generally perceived physicians’ current digital skills as “good” or “very good” (417/524, 79.6%). However, when asked where medical education should primarily focus assuming reliable AI for technical tasks, a clear majority (368/524, 70.2%; 95% CI 66.3%‐74.1%) prioritized human-centered skills such as empathy, communication, and ethical judgment. When reflecting on consultations in which they had discussed AI-derived health information, most participants recalled collaborative or positive physician reactions (305/524, 58.2%; 95% CI 54.0%‐62.4%), with overtly dismissive or defensive responses described only by a small minority.

These findings suggest that, while the public expresses significant caution and specific concerns regarding autonomous AI diagnostics, there is a clear preference for AI to be integrated as a supportive, physician-led tool rather than being rejected entirely. The data reflect a nuanced “guarded acceptance,” where trust is contingent on human oversight and formal medical training.

### Public Trust in Diagnostic Scenarios and Preference for Physician-Led Care

Our diagnostic scenario findings dovetail with experimental work showing that people remain cautious about AI when it is introduced into core clinical decision-making. In a randomized online experiment with 1762 American adults, Chen and Cui [2] found that simply describing a physician as using AI assistance reduced both trust and intention to seek care compared with identical vignettes that either did not mention AI or explicitly stated that the physician did not use AI. On a scale from 0 to 1, mean trust and help-seeking scores were highest when AI was explicitly absent and lowest when AI was used “extensively,” with large negative effects of AI use on intention to seek care. Previous behavioral work by Longoni et al [14] also demonstrated that patients may avoid AI even when its performance is presented as equivalent to that of a physician: in one experiment, 40% of participants enrolled when a free stress diagnosis was described as being performed by a human clinician, but only 26% enrolled when the same service was described as being provided by a computer despite identical accuracy information across conditions.

Experimental data on human-AI collaboration further support the idea that the public prefers physician-led models where AI acts as an adjunct rather than a primary decision-maker. In an online study of patients in the United States, Esmaeilzadeh et al [15] compared scenarios in which diagnoses were provided by AI alone, a physician alone, or a physician collaborating with AI. On a trust scale from 5 to 25, marginal mean trust scores were lowest for AI-only scenarios (16.0) followed by AI and physician scenarios (17.1) and were highest for physician-only scenarios (18.4). Our finding that respondents trusted a physician-only diagnosis the most (329/524, 62.8%; 95% CI 58.6%‐66.9%) followed by a physician-first-then-AI model (140/524, 26.7%; 95% CI 22.9%‐30.5%) while showing little enthusiasm for AI-first scenarios (41/524, 7.8%; 95% CI 5.5%‐10.1%) is consistent with these results.

Multinational patient surveys also show a similar structure of attitudes. In a large study of 13,806 hospital patients, Busch et al [16] reported that 57.6% of respondents held a positive view of AI in health care overall, yet less than half (43.6%) trusted AI to provide reliable diagnostic information. Only 4.4% preferred fully autonomous AI systems; in contrast, most wanted physician-led decision-making.

### Expectations for Training, Governance, and Avoidance of Overreliance

Participants in this survey linked their trust in clinicians who use AI to explicit, formal training on the limitations and potential errors of AI systems. Nearly 60% (311/524, 59.4%; 95% CI 55.1%‐63.6%) rated such training as “essential,” and a further 34.5% (181/524; 95% CI 30.5%‐38.6%) rated it as “very important.” This demand is likely driven by the widespread concern that future physicians might rely too much on AI, with 81.1% (425/524) of our sample being “very concerned” (134/524, 25.6%; 95% CI 21.8%‐29.3%) or “extremely concerned” (291/524, 55.5%; 95% CI 51.3%‐59.8%) about this issue. These expectations mirror themes identified in qualitative work where participants stressed worries about safety and bias, emphasizing that clinicians should act as safeguards who check and contextualize AI outputs [17].

### Support for Mandatory AI Education in Medical Schools

The high level of agreement in our sample regarding AI-related training aligns with expectations observed in surveys of health profession students. In Canada, Teng et al [11] found that 78.77% of students expected AI to affect their careers. A similar pattern can be found among American medical students; Liu et al [8] reported that 91.5% agreed that training on AI would be useful. However, only 8.8% indicated that their schools provided such resources, and only 8.7% had any formal training on AI.

Our survey did not ask respondents to design curricula, but the finding that 85.1% (446/524) agreed that training on AI use, ethics, and limitations should be mandatory, with 61.5% (322/524; 95% CI 57.3%‐65.6%) “strongly” agreeing and 23.7% (124/524; 95% CI 20.0%‐27.3%) agreeing, suggests that public expectations and student aspirations are aligned. This convergence suggests a shared expectation between future professionals and the public that AI topics should be formally addressed in curricula. Our findings suggest that public support for AI education is not necessarily an endorsement of AI-driven care itself but rather a demand for risk mitigation. Just as patients in acute settings, such as the emergency department, may prefer human-only care but accept technology if it improves efficiency, they demand that such use be strictly supervised. Consequently, the public views training on AI as an essential safeguard; they want physicians to be “AI literate” specifically to understand the limitations of these tools and limit potential harms. This shifts the narrative of medical education from simply “learning to use AI” to “learning to safely oversee AI.” This highlights an ongoing conversation for medical educators regarding how to best integrate AI literacy without compromising existing clinical training. This does not necessarily require entirely new departments. AI can be integrated as a tool for “learning support” and “personalized learning pathways” [18]. Practical models for this implementation are already emerging. For example, a web-based, self-guided elective course for senior medical students, which focused on dataset analysis and project-based learning, demonstrated an increase in self-reported confidence in AI skills and received high qualitative satisfaction ratings from participants [12]. Such models provide a feasible blueprint for developing the critical appraisal and technical understanding that our findings show the public expects from future physicians.

### Digital Skills and the Call for Human-Centered Training Priorities

Respondents in this study tended to view physicians as already reasonably digitally competent. Nearly 80% (417/524, 79.6%) rated physicians’ digital skills as “good” (156/524, 29.8%; 95% CI 25.9%‐33.7%) or “very good” (261/524, 49.8%; 95% CI 45.5%‐54.1%). Nevertheless, when asked to identify where medical education should place its primary focus if AI reliably handles more technical tasks, a clear majority (368/524, 70.2%; 95% CI 66.3%‐74.1%) favored human-centered skills—empathy, patient communication, and ethical judgment—over core clinical procedures (156/524, 29.8%; 95% CI 25.9%‐33.7%).

This preference aligns with a systematic review that found that participants consistently voiced concerns about loss of human contact and expressed a preference for AI systems operating under human oversight [19]. Our findings suggest that the public does not see digital skills and human-centered skills as competing priorities; rather, they appear to treat digital competence as a given and want any “freed capacity” from AI to be reinvested in the human aspects of care.

### Patient-Physician Interactions Regarding AI-Derived Information

An additional contribution of this study is the focus on patient-reported experiences. Among respondents who recalled such interactions, most described their physicians’ reactions as collaborative or positive (305/524, 58.2%; 95% CI 54.0%‐62.4%), with roughly one-quarter (128/524, 24.4%; 95% CI 20.7%‐28.1%) describing a neutral reaction. Only small minorities reported dismissive or defensive responses. This suggests that, at least in this online US sample, many clinicians may already be engaging with AI-derived information in ways that align with patient expectations for partnership, providing a strong foundation for the human-centered model of care that our respondents prioritized.

### Limitations

Several limitations must be considered. First, this study used a nonprobability convenience sample recruited via SurveyMonkey Audience and is not statistically representative of the general US population. Respondents were predominantly male (340/520, 65.4%) and more often reported higher income brackets. Additionally, the youngest adult demographic (18‐29 years) was notably underrepresented (23/520, 4.4%), which is a relevant limitation as younger, potentially more “AI-native” populations might hold different perspectives on automated diagnostics. Finally, almost all participants completed the survey on mobile devices (499/520, 96.0%), which may indicate a group that is generally more digitally engaged.

Second, all measures were self-reported attitudes collected in hypothetical scenarios. Expressed trust, concerns, and preferences may not fully predict actual behavior. Recall of physician reactions is also subject to memory and social desirability bias.

Third, the survey instrument was intentionally concise. We did not distinguish between different types of AI systems (eg, imaging algorithms and generative chatbots) or different clinical contexts, nor did we collect qualitative data.

Specifically, we must explicitly acknowledge that certain terms used in the survey, such as “complex diagnosis,” were not clinically defined. While this phrasing was intended to convey a high-stakes health scenario to a lay audience without using medical jargon, it is highly subjective. Because we do not know what specific medical scenario the respondents were imagining, their reported levels of trust must be interpreted with caution. For instance, had respondents envisioned a life-or-death emergency (eg, acute trauma), their willingness to accept AI involvement even merely as a “second opinion” might have been substantially lower than if they had imagined a chronic disease diagnostic dilemma. Additionally, public support for mandatory training on AI may be inflated as the survey did not present the zero-sum trade-offs inherent in crowded medical curricula, where adding new topics requires sacrificing existing clinical training.

Finally, the framing of specific survey items may have introduced a priming effect. For example, prefacing a question with the assumption that AI will “reliably handle more technical tasks” could have inadvertently biased respondents toward a more optimistic view of AI’s capabilities, potentially influencing their responses to other items. This optimistic phrasing contrasts with the complex reality of real-world AI deployment. For instance, despite immense initial optimism regarding AI’s ability to automate technical tasks such as analyzing medical imaging, comprehensive reviews have demonstrated that hundreds of predictive deep learning models developed for COVID-19 scan analysis failed clinical validation due to severe methodological flaws and lack of real-world generalizability [20]. Given these clinical realities and this rapidly evolving technological environment, our results should be interpreted as a time-bound snapshot. Replication in more diverse samples will be important.

### Conclusions

Our study reveals that public acceptance of AI in medicine is not a blanket endorsement of automation but rather a “guarded acceptance” deeply contingent on human accountability. The findings suggest that, as AI becomes more prevalent, the value of the human-centered elements of care such as empathy, ethical judgment, and communication will not diminish but will likely become the primary benchmark of quality for patients.

Medical education must evolve beyond technical AI literacy. There is a clear public mandate to frame training on AI as a risk mitigation strategy, ensuring that physicians act as rigorous safeguards against algorithmic error rather than mere users. For policy and practice, this means that the integration of AI must be transparent and physician led. Any attempt to bypass human oversight in complex diagnostics may risk eroding the foundational trust of the patient-physician relationship. Ultimately, the successful deployment of AI in health care will depend less on the sophistication of the algorithms and more on the demonstrated competence of the clinicians who oversee them.

## Abbreviations

AI: artificial intelligence

## References

[1] Beets B, Newman TP, Howell EL, Bao L, Yang S (2023). Surveying public perceptions of artificial intelligence in health care in the United States: systematic review. J Med Internet Res.

[2] Chen C, Cui Z (2025). Impact of AI-assisted diagnosis on American patients’ trust in and intention to seek help from health care professionals: randomized, web-based survey experiment. J Med Internet Res.

[3] Robertson C, Woods A, Bergstrand K, Findley J, Balser C, Slepian MJ (2023). Diverse patients’ attitudes towards artificial intelligence (AI) in diagnosis. PLOS Digit Health.

[4] Kühne S, Jacobsen J, Legewie N, Dollmann J (2025). Attitudes toward AI usage in patient health care: evidence from a population survey vignette experiment. J Med Internet Res.

[5] Gundlack J, Thiel C, Negash S (2025). Patients’ perceptions of artificial intelligence acceptance, challenges, and use in medical care: qualitative study. J Med Internet Res.

[6] Nare M, Jurewicz K (2024). Assessing patient trust in automation in health care systems: within-subjects experimental study. JMIR Hum Factors.

[7] Boillat T, Nawaz FA, Rivas H (2022). Readiness to embrace artificial intelligence among medical doctors and students: questionnaire-based study. JMIR Med Educ.

[8] Liu DS, Sawyer J, Luna A (2022). Perceptions of US medical students on artificial intelligence in medicine: mixed methods survey study. JMIR Med Educ.

[9] Ichikawa T, Olsen E, Vinod A (2025). Generative artificial intelligence in medical education-policies and training at US osteopathic medical schools: descriptive cross-sectional survey. JMIR Med Educ.

[10] Izquierdo-Condoy JS, Arias-Intriago M, Tello-De-la-Torre A, Busch F, Ortiz-Prado E (2025). Generative artificial intelligence in medical education: enhancing critical thinking or undermining cognitive autonomy?. J Med Internet Res.

[11] Teng M, Singla R, Yau O (2022). Health care students’ perspectives on artificial intelligence: countrywide survey in Canada. JMIR Med Educ.

[12] Abid A, Murugan A, Banerjee I, Purkayastha S, Trivedi H, Gichoya J (2024). AI education for fourth-year medical students: two-year experience of a web-based, self-guided curriculum and mixed methods study. JMIR Med Educ.

[13] Application documents according to section 15 BO. Ethics Committee of the Bavarian State Medical Association.

[14] Longoni C, Bonezzi A, Morewedge CK (2019). Resistance to medical artificial intelligence. J Consum Res.

[15] Esmaeilzadeh P, Mirzaei T, Dharanikota S (2021). Patients’ perceptions toward human-artificial intelligence interaction in health care: experimental study. J Med Internet Res.

[16] Busch F, Hoffmann L, Xu L (2025). Multinational attitudes toward AI in health care and diagnostics among hospital patients. JAMA Netw Open.

[17] Richardson JP, Smith C, Curtis S (2021). Patient apprehensions about the use of artificial intelligence in healthcare. NPJ Digit Med.

[18] Chan KS, Zary N (2019). Applications and challenges of implementing artificial intelligence in medical education: integrative review. JMIR Med Educ.

[19] Young AT, Amara D, Bhattacharya A, Wei ML (2021). Patient and general public attitudes towards clinical artificial intelligence: a mixed methods systematic review. Lancet Digit Health.

[20] Roberts M, Driggs D, Thorpe M (2021). Common pitfalls and recommendations for using machine learning to detect and prognosticate for COVID-19 using chest radiographs and CT scans. Nat Mach Intell.

